# Right trace wrong place: a normal capnography trace despite the tip of the tracheal tube existing outside the airway

**DOI:** 10.1002/anr3.12313

**Published:** 2024-07-10

**Authors:** A. Karmakar, M. J. Khan, N. A. H. Shallik, A. H. M. N. Moustafa, Y. M. R. A. Toble, G. F. Strandvik

**Affiliations:** ^1^ Department of Anesthesiology, ICU and Perioperative Medicine Hamad Medical Corporation Doha Qatar; ^2^ Department of Clinical Anesthesiology Weill Cornell Medicine – Qatar Doha Qatar; ^3^ Department of Clinical Anesthesiology, College of Medicine Qatar University Doha Qatar; ^4^ Anesthesia and Surgical Critical Care Department Tanta University Tanta Egypt; ^5^ Department of Diagnostic Radiology and Medical Imaging Hamad Medical Corporation Doha Qatar; ^6^ Department of Trauma Intensive Care Unit Hamad Medical Corporation Doha Qatar

**Keywords:** airway management, capnography, tracheal injury, tracheal intubation

## Abstract

Head and neck trauma can result in difficult airway management. A 25‐year‐old male required emergency tracheal intubation on arrival to the emergency department following a motorbike accident. Despite the presence of a normal capnography a computed tomography scan demonstrated a tracheal opening, an extra‐tracheal position of the distal end of the tracheal tube, and extensive subcutaneous emphysema. The tube was re‐directed into the trachea and the tracheal injury was surgically repaired. This case highlights that the presence of a normal capnograph does not necessarily mean that the distal end of the tracheal tube resides within the airway.

## Introduction

Continuous waveform capnography or ‘sustained exhaled carbon dioxide’ has been recommended to confirm alveolar ventilation after tracheal tube placement. The Project For Universal Management of Airways (PUMA) describes four characteristics which need to be fulfilled: (1) Amplitude rise during exhalation and fall during inspiration; (2) Consistent or increasing amplitude over at least seven breaths; (3) Peak amplitude more than 1 kPa (7.5 mmHg) above baseline; and (4) Reading is clinically appropriate [1]. We describe the case of a polytrauma patient who fulfilled these criteria but where the tracheal tube tip was located in the anterior neck having passed through the anterior tracheal wall.

## Report

A 25‐year‐old male motorcyclist collided with a traffic bollard and was ejected over the handle bars. He was brought by ambulance to the emergency department. On arrival, the patient was alert and wearing a cervical collar which was applied at the scene. There were abrasions on his forehead and both wrists as well as lacerations to both knees without evidence of truncal injury. Neck examination was obscured by the rigid collar, but the visible portion of the neck and jaw appeared swollen. As the patient was vocalising, treating clinicians were reassured the airway was patent and focused examination on distracting injuries. No abnormal airway sounds were appreciated. The patient's vital signs were: blood pressure 190/103 mmHg, heart rate 100 beats.min^−1^, respiratory rate 35 breaths.min^−1^, SpO_2_ 95% receiving 15 l.min^−1^ of oxygen via a non‐rebreather mask.

A chest radiograph demonstrated bilateral pneumothoraces. Peripheral oxygen saturations declined to 68% within minutes of arrival. Thoracostomy drains were inserted bilaterally under local anaesthesia, with confirmation of air release in the underwater seal on both sides. Despite this, there was further rapid decline in SpO_2_ to 27% and the patient became unresponsive. Emergency tracheal intubation was initiated. The cervical collar was temporarily removed but the neck was maintained in a neutral position. An intubating stylet (size 14 Fr EasySeal™ Intubation Stylet, Non‐Change Enterprise Co Ltd, New Taipei City, Taiwan) was inserted into a standard 7.5 mm ID tracheal tube (Shiley™ TaperGuard Evac Oral Tracheal Tube Murphy Eye, Covidien, USA). A stylet is used as default for anticipated difficult airway management in this institution. The stylet was hooked at the proximal end of the tracheal tube to ensure it did not protrude beyond the end of the tube.

Tracheal intubation was initially attempted without drug assistance due to concerns regarding imminent haemodynamic collapse. A grade 3 Cormack‐Lehane view was obtained on direct laryngoscopy due to oropharyngeal oedema, presence of blood and secretions in the mouth and restricted mouth opening from increased muscle tone as the patient started to regain consciousness. After administering 100 mg ketamine and 100 mg rocuronium and suctioning of the oral cavity, a grade 2 Cormack‐Lehane view was obtained. The tip of the tracheal tube, with the stylet contained within it, was passed through the glottis. The stylet was removed after a short portion of the tube passed into the trachea. The tube was then advanced further until it reached a depth of 22 cm at the patient's teeth.

After a normal capnogram was observed with manual ventilation, continuous mandatory ventilation was initiated with the following settings: tidal volume 450 ml; rate 24 breaths.min^−1^, I:E ratio 1:2.8; PEEP 5 cmH_2_O; FiO_2_ 1.0. Bilateral chest expansion was visible and SpO_2_ rapidly improved to 91%. The capnography trace remained normal in nature but was high in amplitude (end‐tidal CO_2_ of 6.8 kPa). The dialled tidal volume was being delivered successfully. The peak inspiratory pressure was 34 cm H_2_O. The tracheal tube was fixed at a depth of 22 cm at the patient's teeth.

**Figure 1:**
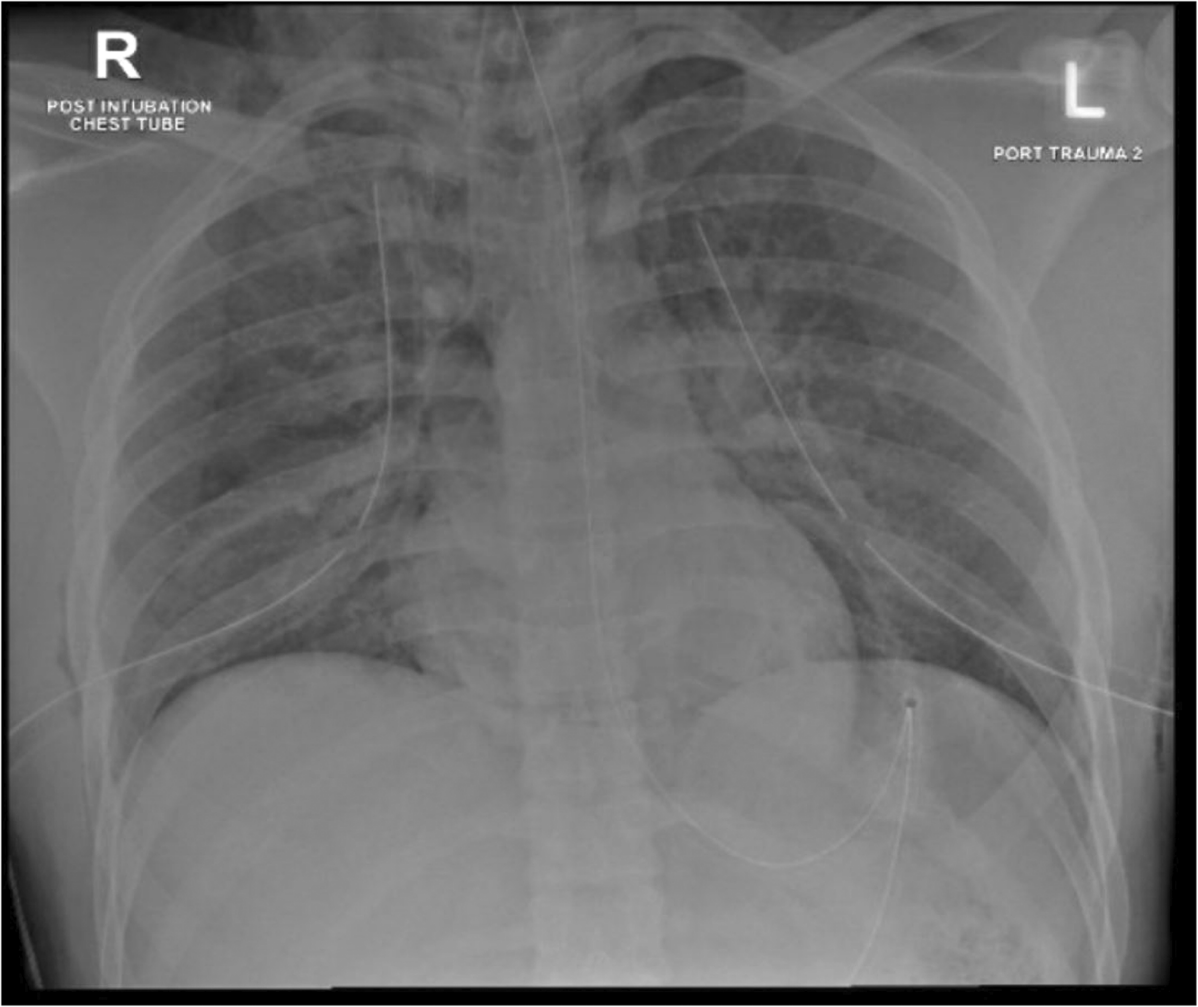
Chest radiograph after tracheal intubation and insertion of bilateral chest drains.

**Figure 2:**
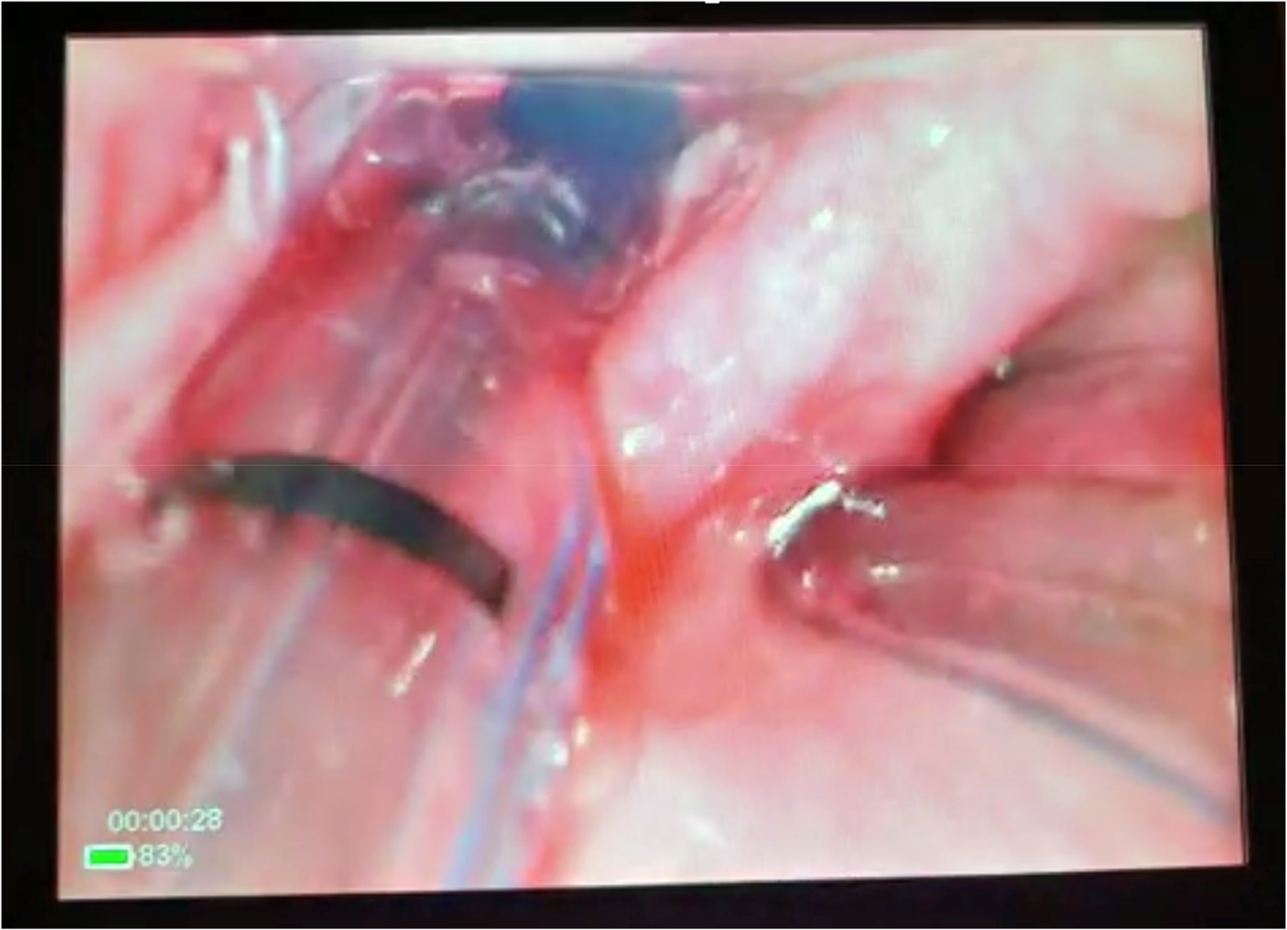
Image obtained on videolaryngoscopy demonstrating passage of the tracheal tube through the vocal cords, with visible black depth markers and an inflated blue cuff. An orogastric tube is also present.

The chest radiograph showed re‐expansion of the lungs with correctly placed chest drains bilaterally (Fig. 1). However, the tracheal tube was not clearly visible on the radiograph. The tube was advanced by 1 cm to a depth of 23 cm, but a repeat chest radiograph did not clearly show the tube. The tube was advanced by a further 3 cm to 26 cm at the teeth but remained difficult to appreciate on a chest radiograph. At this point, videolaryngoscopy was performed with a C‐Mac D‐Blade (Karl Storz, Tuttlingen, Germany) which showed the tracheal tube passing through the glottis (Fig. 2). The tube was felt to have curved in the pharynx with the above attempts at advancement rather than to have passed further into the trachea. A 5.0 mm flexible scope was passed through the tracheal tube to investigate further (Supporting Information, Video S1). The distal end of the tracheal tube appeared to be occluded by tissue and the carina could not be visualised. The Murphy eye was patent.

**Figure 3:**
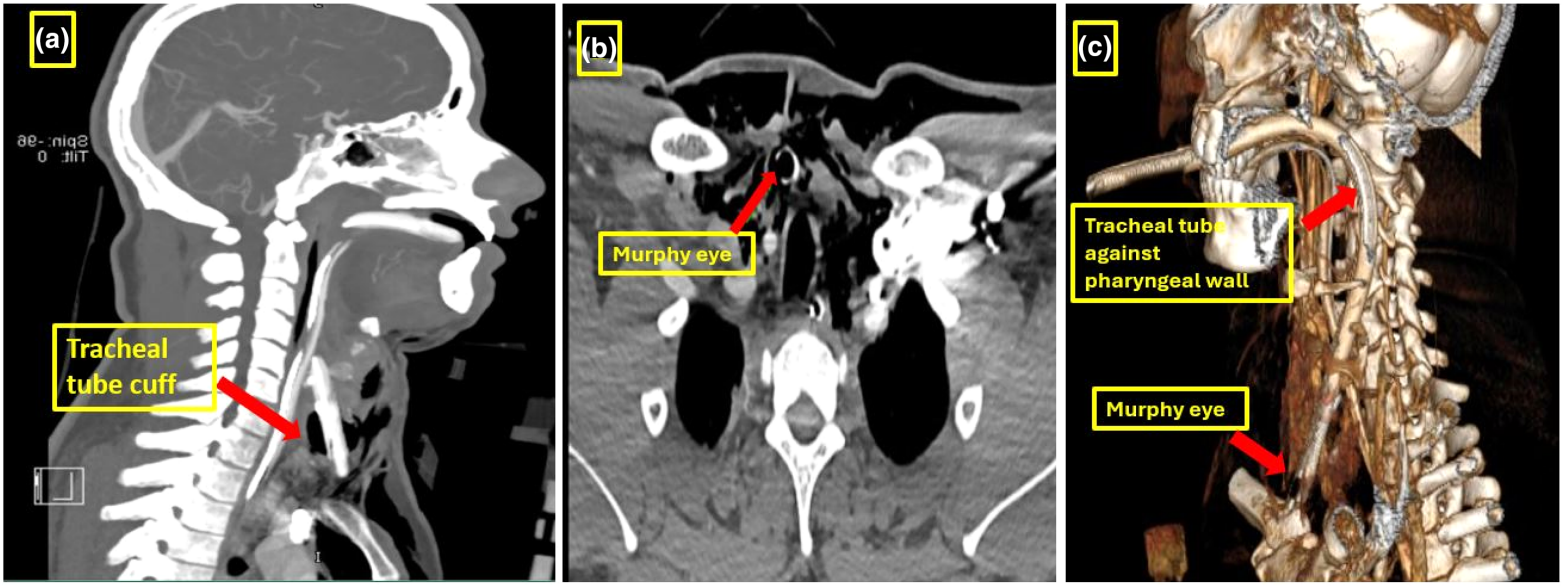
Computed tomography images showing (a) passage of the distal end of the tracheal tube into the anterior neck with inflated cuff and significant emphysema (b) location of the Murphy eye outside the tracheal lumen (c) a tracheal tube taking a curved course and lying against the posterior wall of the pharynx when known to be at a depth of 26 cm.

As oxygenation and ventilation were adequate despite concerns about airway position, a whole‐body computed tomography scan was completed to investigate for the presence of life‐threatening injuries. This showed extensive emphysema of the neck and chest. The tip of the tracheal tube resided in the anterior neck, having passed through the anterior wall of the trachea (Fig. 3; Supporting Information, Video S2). The capnography trace remained normal, with stable gas exchange and haemodynamics.

The patient was transferred to the operating room where the on‐call thoracic surgeon, ENT surgeon and ECMO team were on standby during airway management. Using a flexible scope, the tracheal tube was safely withdrawn and its tip was re‐directed into the tracheal lumen. The anterior tracheal wall was surgically repaired.

The patient was admitted to the trauma intensive care unit and was extubated uneventfully 24 h later. He underwent further surgeries for repair of extremity fractures under regional anaesthesia and made an uneventful recovery.

## Discussion

Tracheal injury can result from both blunt and penetrating trauma. It can involve the extra thoracic cervical trachea and the distal intra thoracic trachea and bronchi. A cervical tracheal injury may not be readily apparent but should be considered in any patient with a significant mechanism of injury and injuries to the face and mandible [2, 3]. These patients will often have subcutaneous emphysema, pneumo mediastinum and pneumothorax which act as further clues to suggest tracheobronchial injury [3, 4]. However, subcutaneous emphysema may not develop immediately, or may not be present at all [5].

Airway management in any patient with head, neck or chest trauma can be challenging due to distorted anatomy, presence of blood and secretions, compromised physiology, full stomach status, compromised oxygenation and risk of iatrogenic tracheal injury [6]. It is unclear whether the tracheal injury in this patient was due to the initial trauma or iatrogenic. Since the patient was able to speak, there was no significant concern for an airway injury. However, the presence of neck swelling before intubation could have suggested underlying subcutaneous emphysema. It is also possible that intubation with a styletted tracheal tube resulted in iatrogenic injury. A stylet‐related injury may be preventable by ensuring the stylet does not reach the tip of the tracheal tube, does not itself pass through the vocal cords and is withdrawn as the tube is advanced into the trachea.

Tracheal tube misting, chest rise and lung auscultation are all unreliable methods for confirming correct tracheal tube placement [7]. The phrase ‘no trace wrong place’ reflects the importance of capnography in detecting oesophageal intubation [8]. This case represented a scenario of ‘right trace and wrong place’ as there was a normal capnograph despite the tracheal tube herniated through the anterior tracheal wall. In 1997, Baumgartner and colleagues reported a similar case of adequate ventilation despite an extra‐tracheal position of the tube [9]. All four of the criteria for ‘sustained exhaled carbon dioxide’ recommended by the Project for Universal Management of Airways to confirm correct tracheal tube position were also present in this case [1].

As the end of the tracheal tube appeared occluded, both inspiratory and expiratory gas flow likely occurred through the Murphy eye. Even though the Murphy eye itself was also outside the trachea on imaging, the gas may have passed through this orifice and then backwards along the outside of the tube to enter into the trachea. The cuff of the tracheal tube remained within the proximal trachea, preventing the administered gas from passing into the upper airway and creating a sealed compartment to achieve lung inflation.

This case highlights a rare scenario whereby the tip of a tracheal tube is not located within the airway despite a normal capnography trace and adequate gas exchange. When there is uncertainty regarding tracheal tube position, early use of flexible bronchoscopy can be beneficial for clarifying its location and for enabling safe readjustment, if needed.

## Supporting information


**Video S1.** Passage of a flexible bronchoscope through the tracheal tube. The distal end of the tube is occluded by tissue and the carina is not visualised.
**Video S2.** Computed tomography images of the head, neck and thorax showing the tracheal tube migrating out of the trachea into the anterior neck.
