# Supplementary material for: Right trace wrong place: a normal capnography trace despite the tip of the tracheal tube existing outside the airway
Source: Anaesth Rep. 2024 Jul 10;12(2):e12313. doi: 10.1002/anr3.12313 (PMC11237171; doi:10.1002/anr3.12313)
Supplement: Supplementary file 1 — Video S1. Passage of a flexible bronchoscope through the tracheal tube. The distal end of the tube is occluded by tissue and the carina is not visualised. Video S2. Computed tomography images of the head, neck and thorax showing the tracheal tube migrating out of the trachea into the anterior neck. [file ANR3-12-e12313-s001.pptx]

## Slide 1
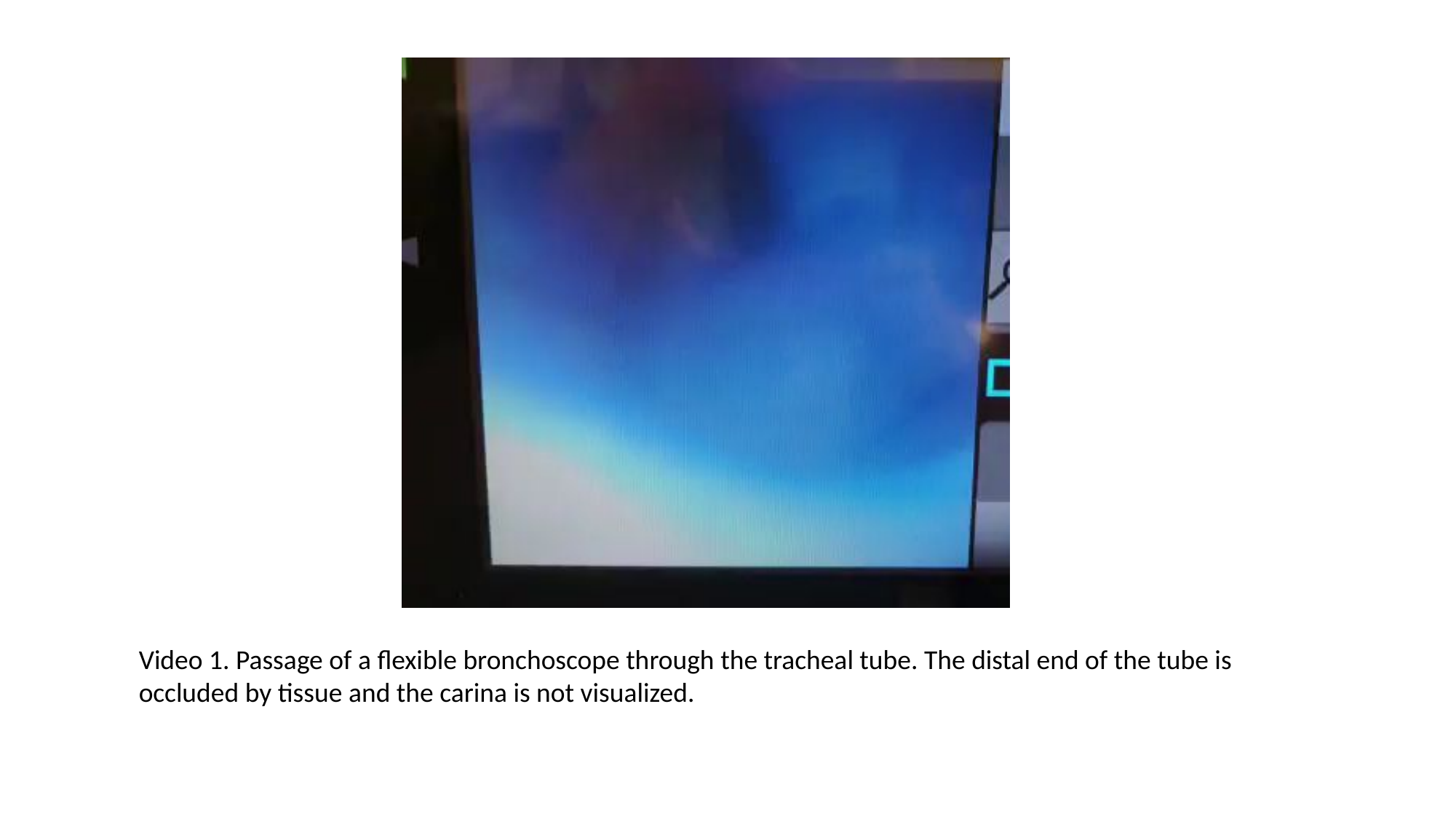

Video 1. Passage of a flexible bronchoscope through the tracheal tube. The distal end of the tube is occluded by tissue and the carina is not visualized.

## Slide 2
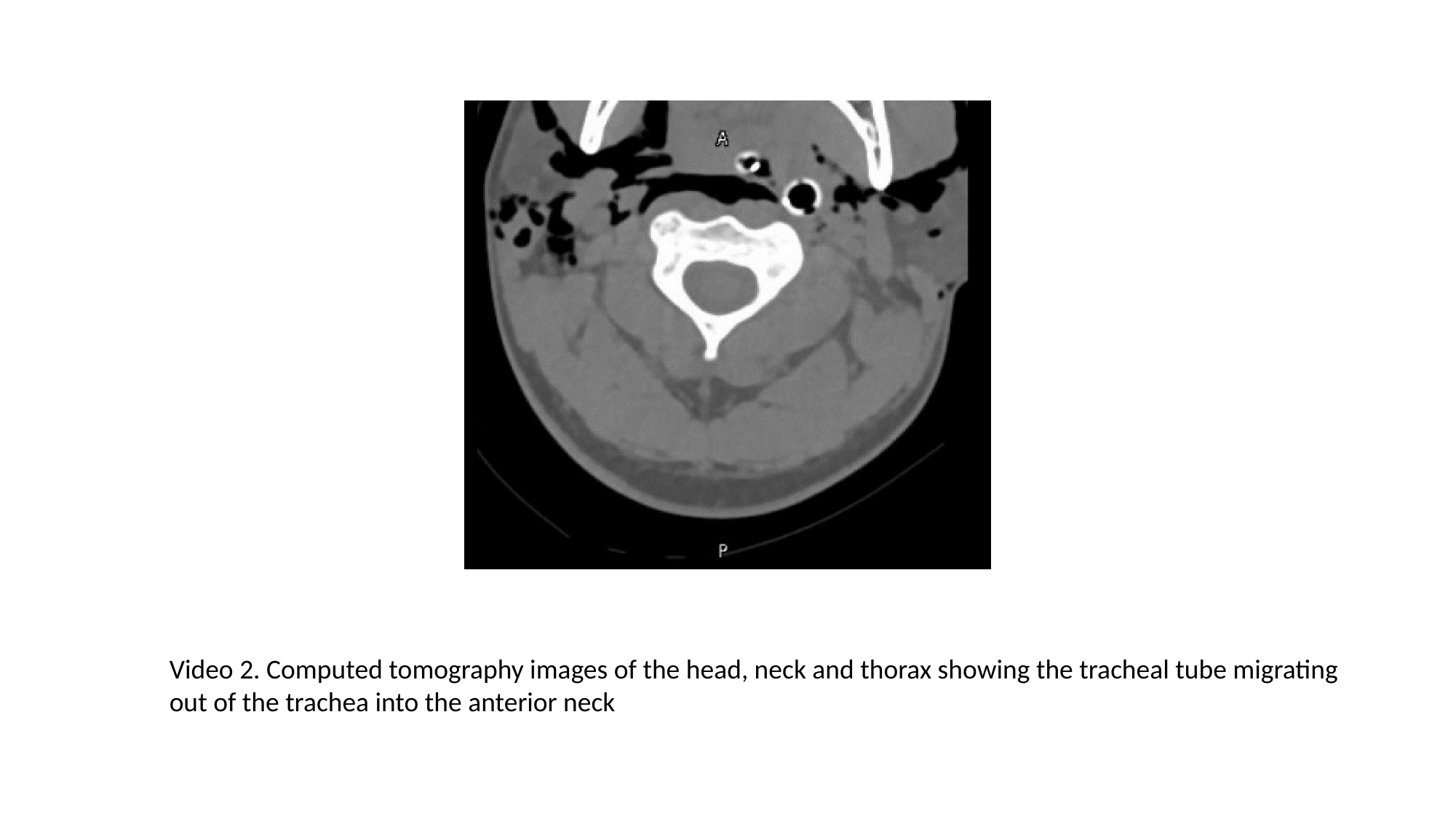

Video 2. Computed tomography images of the head, neck and thorax showing the tracheal tube migrating out of the trachea into the anterior neck
